# Enhanced Insulin Secretion Through Upregulation of Transcription Factors by Hydroalcoholic Extract of *Securigera securidaca* Seeds in Diabetic Animal Model

**DOI:** 10.1002/edm2.515

**Published:** 2024-09-05

**Authors:** Maryam Hasani, Ebrahim Abbasi‐Oshaghi, Fatemeh Khomari, Bahar Kiani, Fatemeh Mirzaei, Iraj Alipourfard, Iraj Khodadadi, Heydar Tayebinia, Mohammad Babaei, Shahin Alizadeh‐Fanalou, Elham Bahreini

**Affiliations:** ^1^ Department of Biochemistry Medical School, Hamadan University of Medical Sciences Hamadan Iran; ^2^ Department of Clinical Biochemistry, School of Medicine Iran University of Medical Sciences Tehran Iran; ^3^ Department of Anatomical Sciences, School of Medicine Hamedan University of Medical Sciences Hamedan Iran; ^4^ University of Silesia Katowice Poland; ^5^ Department of Clinical Sciences, Faculty of Veterinary Science Bu‐Ali Sina University Hamedan Iran; ^6^ Department of Clinical Biochemistry, School of Medicine Urmia University of Medical Sciences Urmia Iran

**Keywords:** diabetes, FGF21, insulin, MafA, Nrf2, PDX1, *Securigera securidaca*

## Abstract

**Aim:**

In previous studies, the researchers observed an increase in insulin secretion in STZ‐treated diabetic rats following treatment with the hydroalcoholic extract of *Securigera securidaca* (HESS) seeds. This study focuses on the relationship between the antioxidant properties of HESS with changes in diabetic pancreatic tissue and the gene expression of factors that impact insulin secretion.

**Methods:**

In this controlled experimental study, three varying doses of HESS were administered to three groups of diabetic rats induced by STZ. Oxidative stress indicators like total antioxidant capacity (TAC), total oxidant status (TOS) and malondialdehyde were assessed in both pancreatic and liver tissues. Pancreatic histology was studied post‐haematoxylin staining. Insulin and FGF21 levels in the blood were measured using the ELISA method. The expression of Nrf2 and FGF21 genes in the pancreas and liver, along with MafA and PDX‐1 genes in the pancreas, was quantified using real‐time PCR.

**Results:**

The administration of HESS in varying doses led to a dose‐dependent rise in blood insulin levels and a decrease in blood glucose levels and oxidative stress. By reducing oxidative stress, HESS treatment lowered the heightened levels of NRF2 and FGF21 in the liver and pancreas of diabetic rats, improving pancreatic tissue health. As oxidative stress decreased, the expression of MafA and PDX1 genes in the pancreas approached levels seen in healthy rats.

**Conclusion:**

HESS elicits an increase in insulin secretion through the mitigation of oxidative stress and tissue damage, as well as the modulation of gene expression related to the insulin transcription factors PDX‐1 and MafA.

## Graphical abstract

In the previous study, researchers observed an increase in insulin secretion in diabetic mice treated with STZ following treatment with hydroalcoholic extract of *Securigera securidaca* (HESS) seeds. This study focused on the relationship between the antioxidant properties of HESS at different doses and changes in the pancreatic tissue of diabetic mice, as well as the expression of genes that affect insulin secretion. It was found that HESS increases insulin secretion by reducing oxidative stress and tissue damage, as well as modulating the expression of genes related to insulin transcription factors PDX‐1 and MafA. It was concluded that *Securigera securidaca* could be considered a good supplement alongside hypoglycemic medications for diabetic patients.

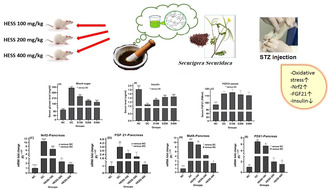

## 1 Introduction

The predominant mechanism of action for many blood glucose‐lowering medications involves either enhancing insulin secretion from the remaining healthy pancreatic beta cells or increasing glucose uptake into cells [1, 2]. Recent attention has been directed towards the utilisation of antidiabetic plants, with *Securigera securidaca* being investigated in a prior study to enhance insulin secretion in streptozotocin (STZ)‐induced diabetic rats [3]. STZ, a cytotoxin derived from the bacterium *Streptomyces achromogenes*, is commonly used to induce experimental type 1 diabetes in rodent models due to its specific targeting of pancreatic beta cells. The study raised inquiries about the mechanism behind the increased insulin secretion by the *Securigera securidaca* plant extract in the context of STZ‐induced damage to pancreatic beta cells. STZ acts as a glucose analogue that selectively accumulates in pancreatic cells containing the glucose transporter GLUT2, affecting beta cells expressing this transporter while sparing other islet cells with GLUT1 transporters [4, 5]. Upon entering beta cells, STZ causes DNA damage through alkylation by its N‐methyl‐N‐nitrosourea component, triggering the activation of poly ADP‐ribose synthetase (PARS) for DNA repair. This repair process depletes cellular NAD+, leading to reduced glycolysis and ATP production. The ensuing decline in ATP levels diverts more substrates to xanthine oxidase, resulting in the production of reactive oxygen species (ROS) like hydrogen peroxide and hydroxyl radicals, inducing oxidative stress. Furthermore, the N‐methyl‐N‐nitrosourea side chains of STZ can release nitric oxide (NO), which disrupts mitochondrial function by inhibiting aconitase activity. Consequently, the cytotoxic impact of STZ on pancreatic beta cells and its role in diabetes induction primarily involve ROS, reactive nitrogen species (RNS) and the initiation of inflammatory responses [5, 6, 7].

β‐cells, crucial for insulin production, are highly metabolically active and heavily rely on oxidative phosphorylation to generate ATP. The process of insulin secretion, particularly in hyperglycaemic conditions, consumes a significant amount of oxygen, making β‐cells susceptible to increased ROS production [2]. ROS, including superoxide anion radical ($O_{2}^{•-}$) and hydrogen peroxide (H_2_O_2_), are generated as by‐products of mitochondrial oxidative phosphorylation. While $O_{2}^{•-}$ is a reactive radical, it can be converted to H_2_O_2_ by superoxide dismutase and further broken down to harmless components like O_2_ and H_2_O by enzymes such as glutathione peroxidase, catalase, and peroxiredoxin. However, due to the limited expression of antioxidant genes, β‐cells are vulnerable to the continuous production of superoxide anions and resultant oxidative stress damage [8, 9, 10].

Oxidative stress‐induced damage to β‐cells results in compromised insulin secretion through various mechanisms. These include reactions of ROS with enzyme thiol groups involved in glycolysis, leading to a decrease in the ATP/ADP ratio, interactions of ROS with potassium channel thiol groups causing their dysfunction, and diminished ATP production due to the oxidation or nitrosylation of mitochondrial complex IV [11]. β‐cells have limited lactate dehydrogenase levels, making it challenging for increased glycolysis to compensate for ATP deficiency. ROS not only hinders insulin secretion but also reduces its gene expression by suppressing the activity of transcription factors like PDX1 and MafA [12]. During stressful conditions, β‐cells activate Nrf2 (nuclear factor erythroid 2‐related factor 2) to maximise their antioxidant defence mechanisms for survival. Under normal circumstances, Nrf2 is bound to Keap1 (Kelch‐like ECH‐associated protein 1)–Cul3–E3 ubiquitin ligase complex in the cytoplasm, where it is swiftly ubiquitinated at lysine residues within the Neh2 domain and degraded in the proteasome. When exposed to oxidative stress, critical cysteine residues, particularly C151 and C288 in the BTB (Bric‐a‐brac, Tramtrack and Broad Complex) domain of Keap1, undergo oxidation upon interaction with electrophiles. This oxidation disrupts the Keap1–Cullin3 complex, releasing Nrf2 from ubiquitination and degradation. Following phosphorylation at Ser40 by PKC and translocation into the nucleus, Nrf2 forms a complex with a small Maf protein. This complex binds to the promoters of genes containing antioxidant response elements (AREs) in their regulatory regions [13, 14].

Fibroblast growth factor 21 (FGF21) is a crucial endocrine regulator impacting energy homeostasis. It is primarily produced in the liver and plays a role in glucose uptake, adipose tissue lipolysis and hepatic ketogenesis. FGF21 is essential for pancreatic β‐cell protection, and its absence can lead to β‐cell failure and reduced insulin secretion. Administering FGF21 in diabetic models has shown benefits in managing hyperglycaemia, hyperlipidaemia and insulin resistance. Studies have indicated that FGF21 influences the expression of transcription factors related to these metabolic processes [15, 16].

*Securigera securidaca* (*S. securidaca*), commonly known as Bitter Lentils, is a potent herb renowned for its anti‐hyperglycaemic, anti‐hyperlipidaemia and anti‐hypertensive properties [17]. Widely utilised in traditional medicine, the hydroalcoholic extract of *S. securidaca* seeds has been analysed using HPLC and GC‐MS, revealing a rich composition of flavonoids, saponins, tannins and alkaloids [18]. Phytochemical examination has identified the significant levels of aromatic compounds, L‐ascorbic acid, dodecanedioic acid derivatives, oxygenated hydrocarbons and β‐sitosterol in this extract. Building on the antioxidant properties previously observed in the hydroalcoholic seed extract of *S. securidaca* (HESS), this study delves into the mechanism by which HESS enhances insulin secretion. Specifically, the research focuses on investigating the expression levels of MafA and PDX‐1, crucial transcription factors involved in insulin secretion, in STZ‐induced diabetic models.

## 2 Materials and Methods

### 2.1 Preparation of the Hydroalcoholic Seed Extract of *S. securidaca*

The hydroalcoholic extract of *S. securidaca* (HESS) was prepared following a methodology partially adapted from a previous study conducted by the authors on the nigella sativa plant [19]. The seeds were processed in accordance with IUCN guidelines (https://portals.iucn.org/library/efiles/documents/PP‐003‐En.pdf) and assigned the herbarium code PMP‐756 by the Medicinal Plants Research Center at the Faculty of Pharmacy, Tehran University of Medical Sciences. The extraction process involved using the mercerisation method with 70% ethanol, followed by concentration through rotary evaporation and storage at 4°C. The total phenolic content of the extract was determined using the Singleton–Rossi colorimetric method with aluminium chloride as a standard [3, 20], while the total flavonoid content was assessed using the Folin–Ciocalteu method with quercetin as a standard reference [3, 21].

### 2.2 Experimental Animal and Study Design

Four‐week‐old male Wistar rats, weighing an average of 230 ± 10 g, were procured from the Experimental Studies Center of Iran University of Medical Sciences. The rats exhibited mean blood glucose levels of 90 ± 6.6 mg/dL as measured with a glucometer. The animal study received approval from the Iran Ministry of Health (Ethic Code: IR.IUMS.FMD.REC.1399.448) and was registered on the Grant No. 17152.

The number of six rats chosen for the study was determined based on statistical power calculation [22]. Following the allocation of six rats to the negative control (NC) group, hyperglycaemia was induced in the remaining rats by intraperitoneal administration of streptozotocin (STZ) at a dose of 55 mg/kg body weight. A blood glucose level equal to or exceeding 250 mg/dL was considered indicative of diabetes. Three days after post‐injection, the diabetic rats were randomly divided into four groups: the diabetic control (DC) group and three groups receiving varying doses of HESS (100, 200 and 400 mg/kg body weight), labelled as E‐100, E‐200 and E‐400 groups, respectively. HESS was orally administered once daily via gavage for 35 days. Overall, the study groups were group 1: negative (or healthy) control, group 2: positive (or diabetic) control, groups 3–5: diabetic groups treated with 100, 200 and 400 mg of HESS per kg body weight, respectively.

### 2.3 Blood Sampling

Following anaesthesia induction in the animals [3], blood samples were collected directly through cardiac puncture. Subsequently, the liver and pancreas were extracted, rinsed with saline and promptly divided into three sections: one segment was preserved in 10% neutral‐buffered formalin for histological analysis, while the remaining two segments were frozen in liquid nitrogen and stored at −80°C for subsequent molecular and biochemical assessments.

### 2.4 Biochemical Analysis

#### 2.4.1 Fasting Glucose and Serum Insulin Measurements

Serum glucose levels were assessed enzymatically using the Pars Azmoon kit (code: P.L:64780179; Iran). Insulin and FGF‐21 levels in the serum were quantified utilising the RayBio Rat Insulin ELISA Kit (code: P01323; United States) and a multiplate ELISA reader (ELISA Reader‐DANA‐320, Japan). The Homeostatic Model Assessment for Insulin Resistance (HOMA‐IR) was calculated using the formula: HOMA‐IR = Insulin (μU/mL) × glucose (nmol/L)/22.5.

Assessment of oxidative stress profiles in liver and pancreatic tissues*Tissue preparation* involved freezing liver and pancreatic tissue in liquid nitrogen and grinding them into a fine powder using a mortar and pestle. A specific quantity of the powdered tissue was homogenised in a predetermined volume of Lysis buffer provided in the kits, followed by centrifugation at 4000 rpm, 4°C for 10 min. The resulting supernatant was utilised for subsequent assays.

*Total tissue antioxidant capacity (TAC)* was assessed using the ferric reducing antioxidant power (FRAP) method in both liver and pancreatic tissues. This method measures the ability of the sample to reduce Fe^3+^ to Fe^2+^, indicating its antioxidant capacity. The reaction between Fe^2+^ and tripyridyltriazine (TPTZ) forms a blue complex with maximum absorption at 593 nm. Serial dilutions of Fe_2_SO_4_ (100–1000 μmol) were prepared in 1 mL of the FRAP reagent provided in the kit (code: Naxifer‐Total Antioxidant Capacity Assay Kit‐TAC; Iran). Standard curves were constructed initially, and TAC values in the samples were determined based on these curves. The results were reported as Fe^2+^ (μmol) per gram of tissue weight [19, 23]. The blood level of TAC was reported as nmol/mL.

*Total tissue oxidant status (TOS)* was determined using a fluorometric method involving 2,7‐dichlorofluorescein diacetate (H_2_DCF‐DA) (code: Navand Salamt, Version 0.52; Iran). H_2_DCF is initially non‐fluorescent but undergoes significant fluorescence upon oxidation to dichlorofluorescein (DCF) in the presence of ROS. In this assay, deacetylated H_2_DCF‐DA is enzymatically converted to DCF by ROS. The fluorescence of DCF was quantified using a Synergy HT Microplate Reader (BioTek Instruments) at 37°C with excitation at 485 nm and emission at 528 nm [19, 24]. The blood level of TOS was reported as nmol/mL.

*Assessment of oxidative stress‐induced damage* involved quantifying malondialdehyde (MDA), a marker of lipid peroxidation (LPO). This evaluation relied on the reaction between 2‐thiobarbituric acid (TBA) and MDA to produce a pink compound with peak absorption at 532 nm according to the kit manufacture's instruction (code: Nalondi‐Lipid Peroxidation Assay Kit‐MDA; Iran). The MDA levels of the tests were determined in comparison with the standard (1,1,3,3‐tetramethoxypropane) prepared in the kit and reported in nmol/mL [19, 25].

#### 2.4.2 Assessment of Gene Expression

The study focused on analysing gene expression levels of Nrf2 and FGF21 in the liver and pancreas, along with PDX1 and MafA in the pancreas, through RT‐qPCR. Tissue samples were processed by crushing in liquid nitrogen and homogenising in TRIZol reagent. RNA extraction and purification were carried out using a total RNA extraction kit (Cat No. YT2551, Yekta Tajhiz Azma; Iran), followed by an assessment of RNA quality and quantity using a NanoDrop instrument and agarose gel electrophoresis. Purified RNA was then converted into single‐strand cDNA using a cDNA synthesis kit (Cat No: YT4500, Yekta Tajhiz Azma; Iran). The reference gene GAPDH was employed for normalisation. Specific primers were designed with the NCBI Primer designing tool, and real‐time PCR was used to amplify both target and reference genes. The gene expression levels were analysed by adjusting the cycle threshold (*C*_*t*_) values and calculating the relative change using the 2^−ΔΔ*Ct*^ method.

### 2.5 Histopathology

Tissues were fixed in 10% neutral‐buffered formalin overnight, followed by dehydration in ethanol, clearing in xylene and infiltration with molten paraffin. The paraffin‐embedded tissues were then thinly sectioned (5–6 μm) using a rotary microtome (Leica RM2255, Germany) and placed on glass slides. After deparaffinisation, the slides underwent staining with haematoxylin and eosin (H&E) stain. Subsequently, a veterinary pathologist examined the slides using a light microscope equipped with a digital camera at 40× and 200× magnification.

### 2.6 Statistical Analysis

Data analysis was conducted with GraphPad Prism9. The Shapiro–Wilk normality test was used to assess data normality. Group differences were evaluated using one‐way ANOVA, followed by Tukey's test for comparing pairs. Results were presented as means ± standard deviation. A *p*‐value below 0.05 was deemed statistically significant.

## 3 Results

### 3.1 Effects of HESS on Insulin and Blood Sugar

**FIGURE 1:**
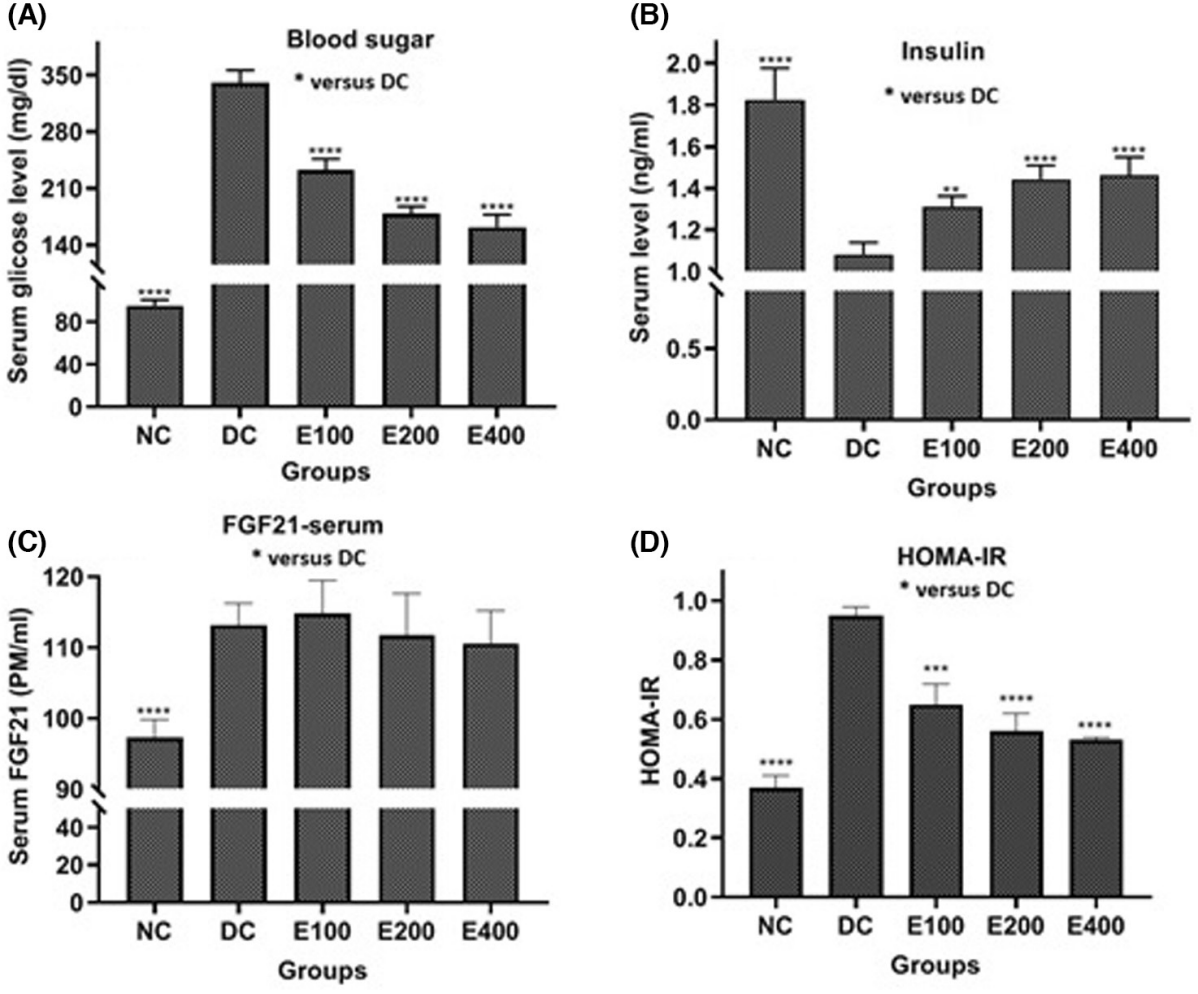
The effect of HESS on the levels of blood glucose (A), insulin (B), FGF21 (C) and HOMA‐IR (D) in the study groups. *Comparison of parameters mentioned above in the ‘NC group’ and ‘HESS treated groups’ with those in the ‘DC group’. *p < 0.05, **p < 0.1, ***p < 0.005, ****p < 0.0001.

As depicted in Figure 1, the effects of HESS on blood glucose (Figure 1A), insulin (Figure 1B), FGF‐21 (Figure 1C) and HOMA‐IR (Figure 1D) were found to be dependent on the dose. Analysis using a one‐way ANOVA test revealed significant differences in the values of these parameters among the groups (*p* < 0.001). Further pairwise comparisons using Tukey's method indicated that HESS led to a significant increase in insulin levels and a decrease in glucose and HOMA‐IR levels (*p* < 0.05) compared to the DC group. However, there was no significant change in serum FGF21 levels across all three treatment groups.

### 3.2 Effects of HESS on Oxidative Stress

Table 1 displays the oxidative stress profiles in the liver and pancreas tissues of the groups under investigation, encompassing TOS, MDA and TAC levels. The results from a one‐way ANOVA analysis demonstrated a notable distinction among the groups for all three factors (*p* < 0.001). Upon conducting pairwise Tukey comparisons between the parameters of the studied groups and those of the DC group, it was observed that elevated TOS and MDA levels, as well as reduced TAC levels in the DC group, were markedly ameliorated by the increased doses of HESS administered in this research.

**TABLE 1:** The effects of HESS on the oxidative stress profile in the liver and pancreas tissues of the study groups.

	Pancreas			Liver		
Parameters	TOS (nmol/mL)	MDA (nmol/mL)	TAC (nmol/mL)	TOS (nmol/mL)	MDA (nmol/mL)	TAC (nmol/mL)
Groups						
NC	3.94 ± 0.1****	10.4 ± 0.8****	5.76 ± 0.3****	5.95 ± 0.5***	0.79 ± 0.05****	2.56 ± 0.11*
DC	5.71 ± 0.3	25.9 ± 0.9	4.04 ± 0.1	8.60 ± 0.3	1.66 ± 0.11	2.00 ± 0.02
HESS‐100	4.99 ± 0.1	25.8 ± 2.1	4.13 ± 0.2	7.52 ± 0.5	1.54 ± 0.06	2.43 ± 0.15
HESS‐200	4.10 ± 0.2	21.1 ± 2.7***	4.74 ± 0.2	7.42 ± 0.2	1.09 ± 0.06***	2.79 ± 0.06***
HESS‐400	3.89 ± 0.2**	15.7 ± 1.6****	5.27 ± 0.3**	6.80 ± 0.1*	0.87 ± 0.08****	2.91 ± 0.11****

*Note:* Comparison of the oxidant profile levels in the liver and pancreas tissues of the ‘NC group’ and ‘HESS‐treated groups’ with those in the ‘DC group.’

Abbreviations: MDA, malondialdehyde; TAC, total antioxidant capacity; TOS, total tissue oxidant status.

*p < 0.05, **p < 0.1, ***p < 0.005, ****p < 0.0001.

### 3.3 Effects of HESS on FGF21 and Nrf2 Gene Expression Levels

**FIGURE 2:**
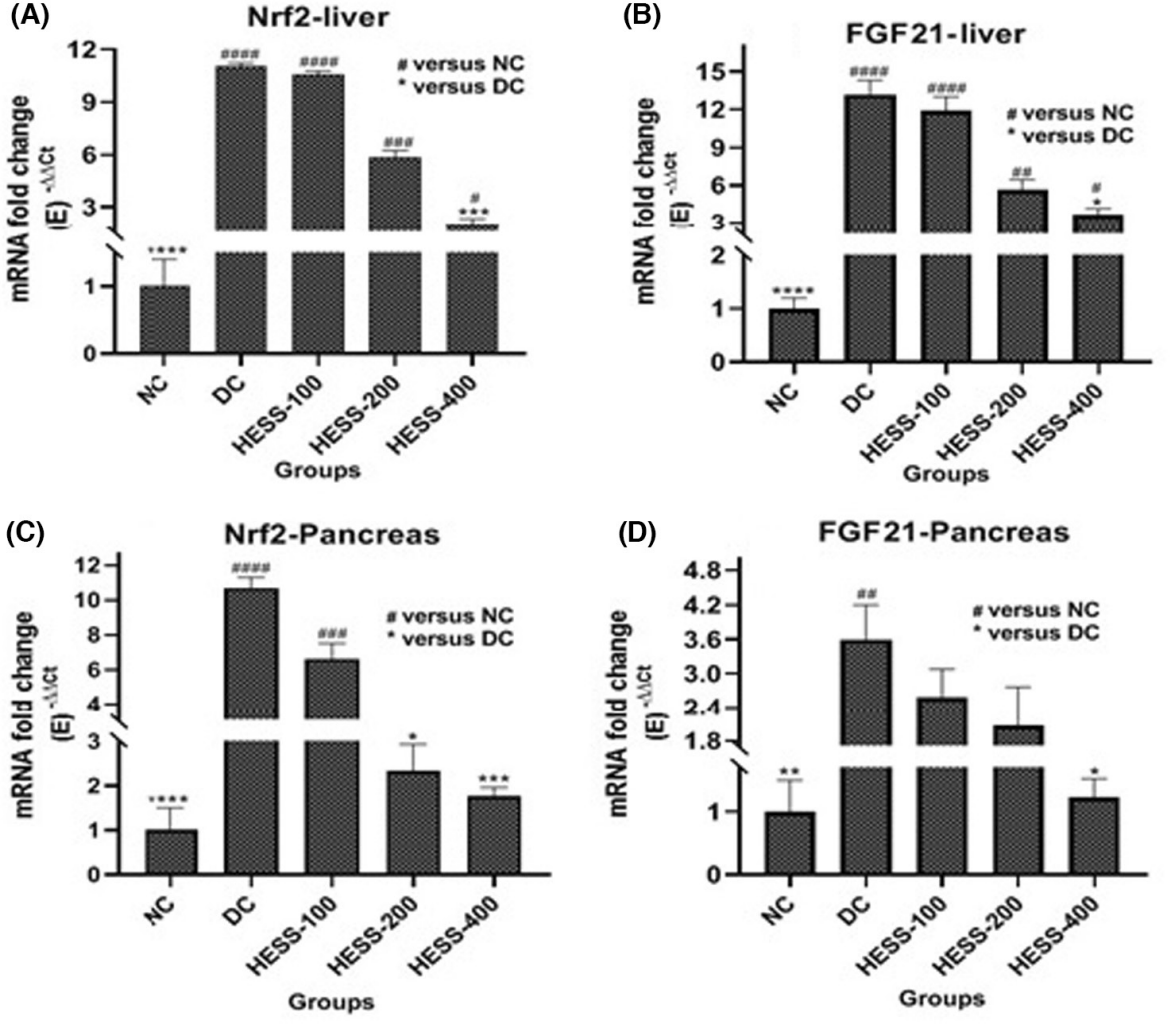
Comparison of mRNA fold changes of gene expression levels of Nrf2‐liver (A), FGF21‐liver (B), Nrf2‐pancreas (C) and FGF‐pancreas (D) in the study groups. *p < 0.05, **p < 0.1, ***p < 0.005, ****p < 0.0001.

Serum FGF21 is predominantly produced by the liver but also acts locally in various tissues as a paracrine factor. This research focused on assessing FGF21 levels in serum and its gene expression in the liver and pancreas. Additionally, the study investigated Nrf2 expression, which is activated during oxidative stress and influences FGF21 gene expression in these organs. Both FGF21 and Nrf2 are pivotal in managing oxidative stress and are crucial for maintaining metabolic balance, especially in regulating glucose metabolism and insulin release. The results depicted in Figure 2 demonstrate a significant increase in Nrf2 and FGF21 gene expression in the liver (Figure 2A,B, respectively) and pancreas (Figure 2C,D, respectively) of diabetic rats compared to healthy controls (*p* < 0.05). While Nrf2 expression rose similarly in both liver and pancreas of diabetic rats, FGF21 expression was notably higher in the liver than in the pancreas. Treatment with HESS led to a dose‐dependent reduction in Nrf2 and FGF21 gene expression in the liver and pancreas of diabetic rats, with statistical significance observed after administering the highest extract dose (*p* < 0.05).

### 3.4 Effect of HESS on Genes Involved in Insulin Production

**FIGURE 3:**
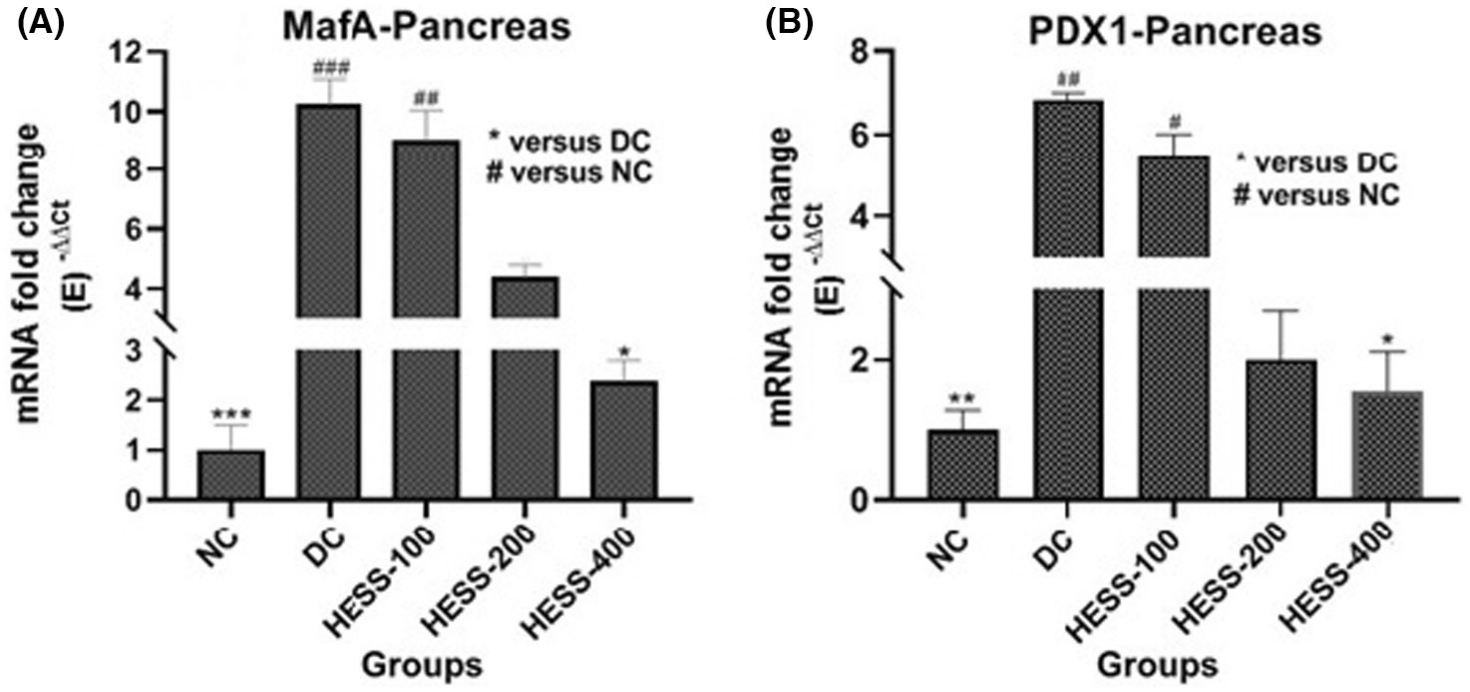
Comparison of mRNA fold changes of PDX1 (A) and MafA (B) gene expression levels in pancreatic tissue in the study groups. *p < 0.05, **p < 0.1, ***p < 0.005.

In the pancreas of the DC group, there was a notable rise in the gene expression of PDX1 (Figure 3A) and MafA (Figure 3B) compared to the NC group (*p* < 0.05). Treatment with HESS in diabetic rats led to a dose‐dependent reduction in PDX1 and MafA levels; nevertheless, this reduction was statistically significant at the highest extract dose (*p* < 0.05).

### 3.5 Effects of HESS on Pancreatic Histology

**FIGURE 4:**
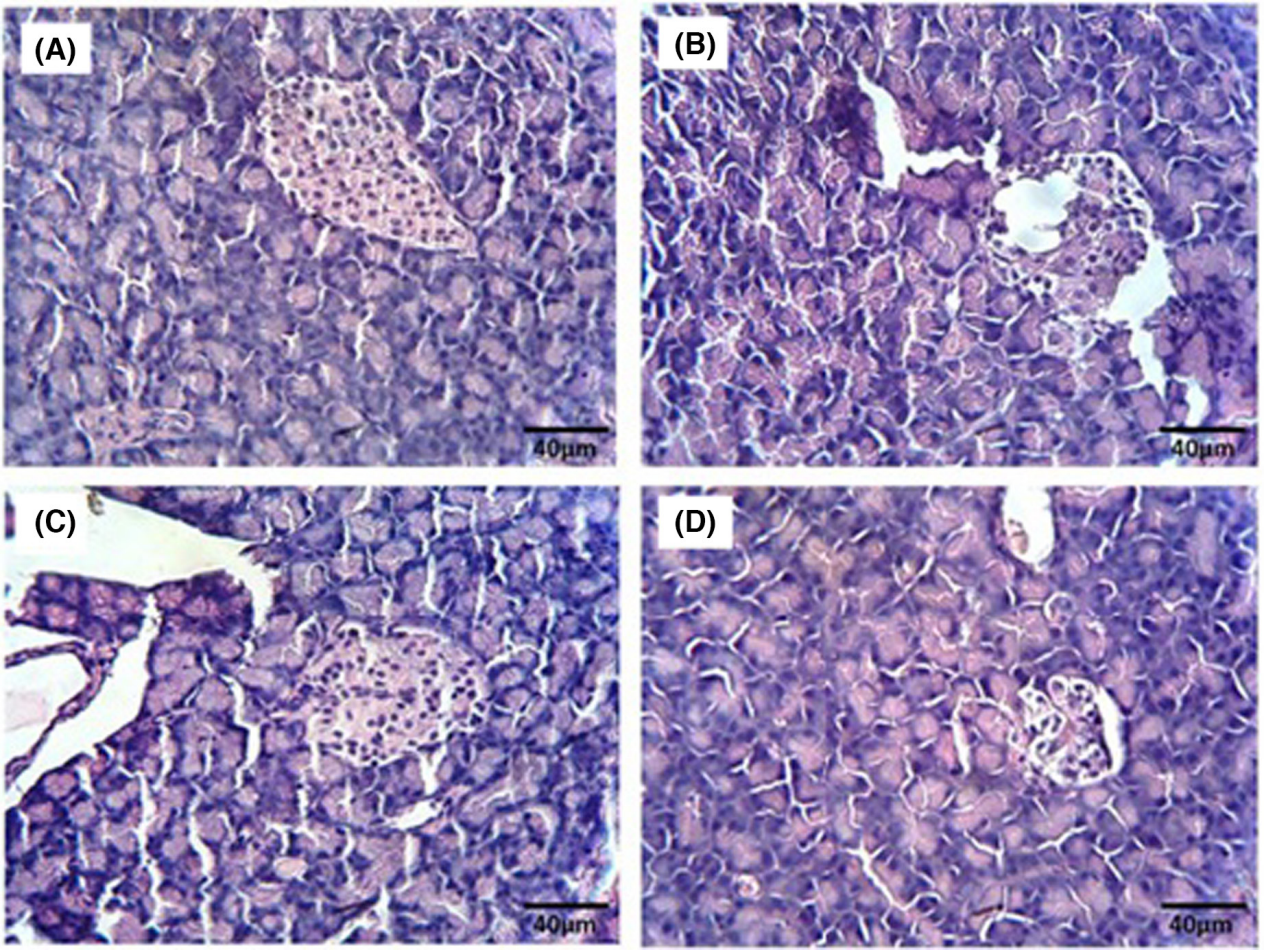
H&E staining and morphological evaluation of pancreatic tissue (Magnification ×100). (A) Normal control group with normal histology. (B) The diabetic control group shows vacuolated cells, an inflamed appearance and decreased cell density. (C) The HESS‐200 group shows a mild decrease in cell density and inflammation in the islets of Langerhans. (D) HESS 400 group with improved histology and normal cell size.

The histological analysis of pancreatic tissue revealed a typical structure in the NC group (Figure 4A). Conversely, examination of pancreatic tissue in the DC group displayed islet dystrophy and cell vacuolation (Figure 4B). Treatment with the extract at doses of 200 and 400 mitigated pancreatic damage in diabetic rats (Figure 4C,D, respectively); notably, at the 400 dose, cell size and tissue structure resembled those of normal tissue.

## 4 Discussion

Similar to our previous study [3], this study also demonstrated a reduction in insulin resistance, repair of damaged beta cells and an increase in insulin secretion in diabetic rats after treatment with HESS. Considering the impact of STZ and the oxidative stress it causes on beta cell damage and reduced insulin secretion [26], and on the other hand, the antioxidant properties of phenolic and flavonoid compounds of *S. securidaca*, this study investigated the effect of the antioxidant properties of *S. securidaca* seeds on genes that stimulate and affect insulin production. Therefore, the aim of this research was to investigate the mechanism of action of *S. securidaca* in improving insulin secretion. For this purpose, the relationship between regulated transcription factors with oxidative stress and factors related to insulin gene activation was evaluated.

The characteristic feature of the antioxidant properties of HESS was the improvement of the oxidative stress profile in diabetic rats [3]. Considering the phenolic and flavonoid content of some plants and their antioxidant properties, especially in eliminating oxidising agents and free radicals [27], the anti‐diabetic property of HESS is likely associated with these compounds [3]. Among the mechanisms mentioned for the anti‐diabetic effects of phenolic and flavonoid derivatives are the inhibition of starch‐digesting enzymes, blocking of sodium‐dependent glucose absorption, stimulation of insulin secretion by beta cells and induction of insulin receptors [28, 29]. Histological study showed severe damage to the pancreatic tissue of diabetic control rats compared to healthy controls. Following treatment of diabetic rats with HESS, it was observed that pancreatic tissue damage improved dose dependently. This tissue improvement can be attributed to a reduction in oxidative stress and an increase in tissue self‐healing power.

In this study, the elevated expression levels of Nrf2 and FGF21 in the liver and pancreas of diabetic desert rats compared to healthy desert rats were associated with increased oxidative stress levels in them. Sun, Huang, and Zhang [30] demonstrated that oxidative stress activates MAPK pathways (JNK and p38), leading to the activation of PKC and PI3K, resulting in phosphorylation and inactivation of Clu3 and Keap1. Nrf2 released in this process enters the nucleus to induce the expression of genes related to antioxidant stress. FGF21 is another target of Nrf2. FGF21 is a factor whose gene is expressed in most tissues, but it plays a more important role in its blood levels in the liver [31, 32]. In the diabetic group, while the gene expression levels of Nrf2 in the liver and pancreas were similar, the gene expression level of FGF21 in the liver was higher than in the pancreas. After treatment with HESS and a reduction in oxidative stress, the expression of Nrf2 and FGF21 in both liver and pancreas tissues, especially in the pancreas, decreased. The increase in serum FGF21 levels in diabetic mice and the subsequent decrease after HESS treatment had a hepatic origin. Pan and colleagues [33] demonstrated the anti‐inflammatory effects of FGF21 on the gastric mucosa and beta cells, but our histological study showed an increase in pancreatic inflammation in desert rats despite the high levels of FGF21. It seems that the improvement of pancreatic damage after HESS treatment is due to the antioxidant properties of HESS without affecting the serum levels of FGF21 [31]. No previous report indicating a direct effect of Nrf2 on insulin transcription factor levels was found, but the interpretation of previous research such as Baumel‐Alterzon et al. [34]. and Suleimanidadan et al. [19] shows that Nrf2 has an indirect effect on positive regulation of insulin secretion. Additionally, Chen et al. reported that the treatment of islet cells from FGF21 knockout mice with FGF21 exogenously increases insulin secretion [35]. Pan et al. also found a positive correlation between high expression of FGF21 in the pancreatic islets of db/db mice, a diabetic mouse model and an increase in insulin transcription factors in the islet cells [33]. They claimed that increased expression of FGF21 in pancreatic beta cells can increase the expression of key transcriptional regulators of insulin production.

MafA and PDX1 are two insulin gene transcription factors, the former exclusively produced by beta cells and the latter produced not only by pancreatic beta cells but also in other non‐beta cells of the pancreas [36]. PDX1 regulates the expression of genes involved in beta‐cell differentiation, insulin production and glucose metabolism. MafA is involved in the maintenance of beta‐cell function and the regulation of insulin secretion. In this study, the observed reduction in the levels of MafA and PDX1 expression in the pancreas of healthy rats (NC group) can be attributed to low blood glucose levels and a high AMP/ATP ratio in beta cells [36]. AMPK is an intracellular energy sensor that becomes activated with an increase in the AMP/ATP ratio, leading to the inhibition of MafA gene expression and insulin secretion [37]. Previous studies have also shown a decrease in the expression of MafA and PDX1 in diabetes [38, 39, 40]. However, contrary to previous studies, the present study demonstrated an increase in the expression of these insulin gene expression stimulating factors in the diabetic control group. Treatment with HESS reduced the expression levels of MafA and PDX1 in diabetic rats, so that the expression levels in the high‐dose extract treatment group were comparable to the healthy control. Furthermore, except for the negative control group, the changes in the expression levels of MafA and PDX1 transcription factors in the study groups were comparable to changes in their blood glucose and insulin levels. Here comes an important question: How can we justify the increase in PDX and MafA levels in controlling diabetes?

Several studies, such as the research conducted by Kondo et al. [41] and El Khattabi and Sharma [42], have shown that chronic hyperglycaemia suppresses AMPK and increases the accumulation of MafA protein, resulting in the positive regulation of GLUT2 and insulin gene expression in beta cells. However, this overwork can eventually destroy the beta cells, especially in the progression from type 2 diabetes to type 1. In the case of STZ treatment, STZ enters beta cells via GLUT2 and induces toxicity by generating reactive oxygen and nitrogen species, which activate MAPK signalling pathways including ERK, JNK and p38 [43]. These MAPK pathways then stimulate the ubiquitination and proteasomal degradation of MafA by altering its phosphorylation pattern, ultimately reducing MafA's DNA‐binding ability [41]. The JNK MAPK pathway prevents insulin from interacting with its receptor and attenuates the PI3K/PKB pathway [44]. This, in turn, stimulates FOXO1 to translocate into the nucleus. While FOXO1 has been described as a negative regulator of PDX1 levels, studies by Kitamura et al. [45] and Zhang et al. [46] have reported that FOXO1 can actually upregulate the expression of NeuroD and MafA. Therefore, the increase in MafA gene expression observed in the diabetic control (DC) group may be attributed to the activation of FOXO1. However, the inability of MafA to effectively promote insulin gene expression can be explained by the changes in MafA's phosphorylation pattern by the ERK and p38 MAPK pathways, although this explanation needs further study.

A rise in blood glucose after a meal triggers the release of insulin from secretory granules into the intercellular space, where it activates the PI3K–PKB pathway through an autocrine effect on the beta cells themselves [47]. The activation of PKB (or Akt) then stimulates insulin gene expression by phosphorylating and inactivating the transcription factor FOXO1. In its active state, FOXO1 forms a complex with Pml and Sirt1 to protect beta cells from oxidative stress by activating the expression of NeuroD and MafA while inactivating PDX1. However, when FOXO1 is phosphorylated, it cannot enter the nucleus, allowing the concentration of PDX1 to increase in the nucleus, thereby activating insulin gene expression [45]. In the case of prolonged hyperglycaemia, the PI3K–PKB signalling pathway becomes impaired due to the phosphorylation of IRS by PKC. Additionally, oxidative stress can oxidise PKB at cysteine residues through H_2_O_2_, rendering the oxidised PKB unable to phosphorylate and inactivate FOXO1 [48]. As a result, the active FOXO1 translocates into the nucleus, where it downregulates PDX1 gene expression by altering the phosphorylation pattern of PDX1. This change in PDX1 phosphorylation leads to its nuclear export, shifting the localisation of PDX1 from the nucleus to the cytoplasm [45, 49]. The expected decrease in PDX1 gene expression in the diabetic control group was not observed in this study. This contradiction may be due to the non‐specificity of PDX1 to beta cells, unlike MafA. PDX1 is expressed not only in beta cells but also in non‐beta cells, particularly under conditions of tissue injury. PDX1 is not only a regulator of insulin gene expression but is also expressed in other cell types beyond just beta cells [12, 49]. It can be inferred that, in the diabetic control group, despite the reduction of PDX1 gene expression by FOXO1 in the beta cells, PDX1 is still expressed in the non‐beta cells of the islets. This may be due to the tissue damage caused by STZ, which triggers the expression of PDX1 in non‐beta cells to facilitate tissue restoration and beta cell differentiation [12]. However, this concept requires a cell line study. The high degree of pancreatic tissue damage observed in the diabetic control group, despite the elevated PDX1 expression, may be attributed to the severity of tissue damage caused by STZ, oxidative stress and hyperglycaemia. In contrast, HESS treatment not only reduced the tissue damage but also regulated the expression PDX1 in proportion to the blood glucose levels. It may be concluded that HESS reduces hyperglycaemia through multiple mechanisms: decreasing oxidative stress, repairing damaged tissues and regulating the expression of PDX1 and MafA. These effects ultimately lead to an increase in insulin production by the beta cells.

## 5 Conclusion

STZ‐induced oxidative stress increased Nrf2 and FGF21 gene expression, but the level of tissue damage exceeded the capacity of the endogenous antioxidant systems to reduce oxidative stress and MDA levels. Despite high PDX‐1 and MafA gene expression, the high glucose and low insulin levels in the diabetic control group were likely due to FOXO1 activation under oxidative stress, which can decrease the activity of these insulin transcription factors. HESS treatment was able to reduce tissue oxidative stress, blood glucose levels and regulate the expression of PDX‐1 and MafA in proportion to the blood glucose levels. These results suggest that the seeds of *S. securidaca* can be considered as a good supplement together with blood sugar‐lowering drugs for diabetics. However, one of the most limitations of this research was that a majority of the result interpretations was based on assumptions and FOXO1 was not evaluated. Therefore, additional investigations on related cell lines are necessary to validate the relationships between the described factors and involved mechanisms.

## Author Contributions

E. Bahraini and E. Abbasi‐Oshaghi designed the project. M. Hasani, F. Khomari, B. Kiani, F. Mirzaei, M. Babaei and Sh Alizadeh‐Fanalou conducted all tests. E. Bahreini analysed the data. E. Bahreini and M.S. Hosseini wrote the main manuscript. E. Bahreini and I. Alipourfard edited the manuscript. I. Khodadadi, H. Tayebinia and other authors reviewed and approved the manuscript.

## Ethics Statement

Animal management was approved by the Ministry of Health of Iran (Ethical code: IR.IUMS.REC.1399.448) and carried out in accordance with the guidelines of the laboratory animal care department of the Iran University of Medical Sciences.

## Conflicts of Interest

The authors declare no conflicts of interest.

## Data Availability

Data presented in this manuscript are available upon request.

## References

[1] A. P. Alves , A. C. Mamede , M. G. Alves , et al., “Glycolysis Inhibition as a Strategy for Hepatocellular Carcinoma Treatment?,” Current Cancer Drug Targets 19, no. 1 (2019): 26–40. 29749314 10.2174/1568009618666180430144441

[2] P. Rorsman and F. M. Ashcroft , “Pancreatic β‐Cell Electrical Activity and Insulin Secretion: Of Mice and Men,” Physiological Reviews 98, no. 1 (2018): 117–214. 29212789 10.1152/physrev.00008.2017 PMC5866358

[3] S. Alizadeh‐Fanalou , M. Babaei , A. Hosseini , et al., “Effects of *Securigera securidaca* Seed Extract in Combination With Glibenclamide on Antioxidant Capacity, Fibroblast Growth Factor 21 and Insulin Resistance in Hyperglycemic Rats,” Journal of Ethnopharmacology 248 (2020): 112331. 31655149 10.1016/j.jep.2019.112331

[4] A. Nahdi , A. John , and H. Raza , “Elucidation of Molecular Mechanisms of Streptozotocin‐Induced Oxidative Stress, Apoptosis, and Mitochondrial Dysfunction in Rin‐5F Pancreatic β‐Cells,” Oxidative Medicine and Cellular Longevity 2017 (2017): 7054272. 28845214 10.1155/2017/7054272 PMC5563420

[5] M. L. Graham , J. L. Janecek , J. A. Kittredge , B. J. Hering , and H. J. Schuurman , “The Streptozotocin‐Induced Diabetic Nude Mouse Model: Differences Between Animals From Different Sources,” Comparative Medicine 61, no. 4 (2011): 356–360. 22330251 PMC3155402

[6] P. Pacher and C. Szabó , “Role of Poly(ADP‐Ribose) polymerase‐1 Activation in the Pathogenesis of Diabetic Complications: Endothelial Dysfunction, as a Common Underlying Theme,” Antioxidants & Redox Signaling 7, no. 11–12 (2005): 1568–1580. 16356120 10.1089/ars.2005.7.1568 PMC2228261

[7] S.‐H. Abtahi‐Evari , M. Shokoohi , A. Abbasi , A. Rajabzade , H. Shoorei , and H. Kalarestaghi , “Protective Effect of Galega Officinalis Extract on Streptozotocin‐Induced Kidney Damage and Biochemical Factor in Diabetic Rats,” Crescent Journal of Medical and Biological Sciences 4 (2017): 108–114.

[8] M. E. Cerf , “Beta Cell Dysfunction and Insulin Resistance,” Frontiers in Endocrinology 4 (2013): 37. 23542897 10.3389/fendo.2013.00037 PMC3608918

[9] U. Asmat , K. Abad , and K. Ismail , “Diabetes Mellitus and Oxidative Stress‐A Concise Review,” Saudi Pharmaceutical Journal 24, no. 5 (2016): 547–553. 27752226 10.1016/j.jsps.2015.03.013 PMC5059829

[10] S.‐H. Abtahi‐Eivari , M. Shokoohi , M. Ghorbani , et al., “Effects of Hydroalcoholic Extracts of Cloves (*Syzygium aromaticum*) on the Serum Biomarkers, Antioxidant Status, and Histopathological Changes of Kidneys in Diabetic Rats,” Crescent Journal of Medical & Biological Sciences 8, no. 4 (2021): 269–275.

[11] D. B. Zorov , M. Juhaszova , and S. J. Sollott , “Mitochondrial Reactive Oxygen Species (ROS) and ROS‐Induced ROS Release,” Physiological Reviews 94, no. 3 (2014): 909–950. 24987008 10.1152/physrev.00026.2013 PMC4101632

[12] Y. Zhu , Q. Liu , Z. Zhou , and Y. Ikeda , “PDX1, Neurogenin‐3, and MAFA: Critical Transcription Regulators for Beta Cell Development and Regeneration,” Stem Cell Research & Therapy 8, no. 1 (2017): 240. 29096722 10.1186/s13287-017-0694-z PMC5667467

[13] D. D. Zhang and M. Hannink , “Distinct Cysteine Residues in Keap1 Are Required for Keap1‐Dependent Ubiquitination of Nrf2 and for Stabilization of Nrf2 by Chemopreventive Agents and Oxidative Stress,” Molecular and Cellular Biology 23, no. 22 (2003): 8137–8151. 14585973 10.1128/MCB.23.22.8137-8151.2003 PMC262403

[14] S. Dayalan Naidu , A. Muramatsu , R. Saito , et al., “C151 in KEAP1 Is the Main Cysteine Sensor for the Cyanoenone Class of NRF2 Activators, Irrespective of Molecular Size or Shape,” Scientific Reports 8, no. 1 (2018): 8037. 29795117 10.1038/s41598-018-26269-9 PMC5966396

[15] Y. Yu , J. He , S. Li , et al., “Fibroblast Growth Factor 21 (FGF21) inhibits Macrophage‐Mediated Inflammation by Activating Nrf2 and Suppressing the NF‐κB Signaling Pathway,” International Immunopharmacology 38 (2016): 144–152. 27276443 10.1016/j.intimp.2016.05.026

[16] Y. Furusawa , A. Uruno , Y. Yagishita , C. Higashi , and M. Yamamoto , “Nrf2 Induces Fibroblast Growth Factor 21 in Diabetic Mice,” Genes to Cells 19, no. 12 (2014): 864–878. 25270507 10.1111/gtc.12186

[17] S. Alizadeh‐Fanalou , A. Nazarizadeh , M. Babaei , M. Khosravi , N. Farahmandian , and E. Bahreini , “Effects of *Securigera securidaca* (L.) Degen & Dorfl Seed Extract Combined With Glibenclamide on paraoxonase1 Activity, Lipid Profile and Peroxidation, and Cardiovascular Risk Indices in Diabetic Rats,” BioImpacts 10, no. 3 (2020): 159–167. 32793438 10.34172/bi.2020.20 PMC7416011

[18] H. K. Aldal'in , G. Al‐Mazaideh , A. H. Al‐Nadaf , et al., “Phytochemical Constituents of *Securigera securidac*a Seed Extract Using GS‐MS and HPLC,” Tropical Journal of Natural Product Research 4, no. 9 (2020): 540–544.

[19] M. Soleimani‐Dodran , R. Alipanah‐Moghadam , F. Jeddi , M. Babaei , R. Salimnejad , and E. Bahreini , “Effect of Hydroalcoholic Seed Extract of Nigella Sativa on Hepatic and Pancreatic Factors of Nrf2 and FGF21 in the Regulation of Insulin Transcription Factors of MafA and PDX‐1 in Streptozotocin‐Treated Diabetic Rats,” Nutrition & Metabolism 19, no. 1 (2022): 64. 36109786 10.1186/s12986-022-00699-9 PMC9479419

[20] S. Chandra , S. Khan , B. Avula , et al., “Assessment of Total Phenolic and Flavonoid Content, Antioxidant Properties, and Yield of Aeroponically and Conventionally Grown Leafy Vegetables and Fruit Crops: A Comparative Study,” Evidence‐Based Complementary and Alternative Medicine 2014 (2014): 253875. 24782905 10.1155/2014/253875 PMC3980857

[21] V. L. Singleton , R. Orthofer , and R. M. Lamuela‐Raventós , “Analysis of Total Phenols and Other Oxidation Substrates and Antioxidants by Means of Folin‐Ciocalteu Reagent,” in Methods in Enzymology. vol. 299 (Cambridge, MA: Academic Press, 1999), 152–178.

[22] X. Zhang and P. Hartmann , “How to Calculate Sample Size in Animal and Human Studies,” Frontiers in Medicine 10 (2023): 1215927. 37663663 10.3389/fmed.2023.1215927 PMC10469945

[23] R. Sudan , M. Bhagat , S. Gupta , J. Singh , and A. Koul , “Iron (FeII) Chelation, Ferric Reducing Antioxidant Power, and Immune Modulating Potential of *Arisaema jacquemontii* (Himalayan Cobra Lily),” BioMed Research International 2014 (2014): 179865. 24895548 10.1155/2014/179865 PMC4033394

[24] C. Y. Hsieh , C. L. Chen , K. C. Yang , C. T. Ma , P. C. Choi , and C. F. Lin , “Detection of Reactive Oxygen Species During the Cell Cycle Under Normal Culture Conditions Using a Modified Fixed‐Sample Staining Method,” Journal of Immunoassay & Immunochemistry 36, no. 2 (2015): 149–161. 24749949 10.1080/15321819.2014.910806

[25] Y. J. Garcia , A. J. Rodríguez‐Malaver , and N. Peñaloza , “Lipid Peroxidation Measurement by Thiobarbituric Acid Assay in Rat Cerebellar Slices,” Journal of Neuroscience Methods 144, no. 1 (2005): 127–135. 15848246 10.1016/j.jneumeth.2004.10.018

[26] H. N. Yousef , S. M. Sakr , and S. A. Sabry , “Mesenchymal Stem Cells Ameliorate Hyperglycemia in Type I Diabetic Developing Male Rats,” Stem Cells International 2022 (2022): 7556278. 35463813 10.1155/2022/7556278 PMC9020910

[27] S. Mathew , T. E. Abraham , and Z. A. Zakaria , “Reactivity of Phenolic Compounds Towards Free Radicals Under in Vitro Conditions,” Journal of Food Science and Technology 52, no. 9 (2015): 5790–5798. 26344993 10.1007/s13197-014-1704-0 PMC4554629

[28] R. K. Al‐Ishaq , M. Abotaleb , P. Kubatka , K. Kajo , and D. Büsselberg , “Flavonoids and Their Anti‐Diabetic Effects: Cellular Mechanisms and Effects to Improve Blood Sugar Levels,” Biomolecules 9, no. 9 (2019): 430. 31480505 10.3390/biom9090430 PMC6769509

[29] G. Williamson and K. Sheedy , “Effects of Polyphenols on Insulin Resistance,” Nutrients 12, no. 10 (2020): 3135. 33066504 10.3390/nu12103135 PMC7602234

[30] Z. Sun , Z. Huang , and D. D. Zhang , “Phosphorylation of Nrf2 at Multiple Sites by MAP Kinases Has a Limited Contribution in Modulating the Nrf2‐Dependent Antioxidant Response,” PLoS One 4, no. 8 (2009): e6588. 19668370 10.1371/journal.pone.0006588 PMC2719090

[31] D. V. Chartoumpekis , P. G. Ziros , A. I. Psyrogiannis , et al., “Nrf2 Represses FGF21 During Long‐Term High‐Fat Diet‐Induced Obesity in Mice,” Diabetes 60, no. 10 (2011): 2465–2473. 21852674 10.2337/db11-0112 PMC3178292

[32] Y. Cheng , J. Zhang , W. Guo , et al., “Up‐Regulation of Nrf2 Is Involved in FGF21‐Mediated Fenofibrate Protection Against Type 1 Diabetic Nephropathy,” Free Radical Biology & Medicine 93 (2016): 94–109. 26849944 10.1016/j.freeradbiomed.2016.02.002 PMC7446394

[33] Y. Pan , B. Wang , J. Zheng , et al., “Pancreatic Fibroblast Growth Factor 21 Protects Against Type 2 Diabetes in Mice by Promoting Insulin Expression and Secretion in a PI3K/Akt Signaling‐Dependent Manner,” Journal of Cellular and Molecular Medicine 23, no. 2 (2019): 1059–1071. 30461198 10.1111/jcmm.14007 PMC6349243

[34] S. Baumel‐Alterzon , L. S. Katz , L. Lambertini , et al., “NRF2 Is Required for Neonatal Mouse Beta Cell Growth by Maintaining Redox Balance and Promoting Mitochondrial Biogenesis and Function,” Diabetologia 67, no. 3 (2024): 547–560. 38206362 10.1007/s00125-023-06071-7 PMC11521447

[35] Z. Chen , L. Yang , Y. Liu , P. Huang , H. Song , and P. Zheng , “The Potential Function and Clinical Application of FGF21 in Metabolic Diseases,” Frontiers in Pharmacology 13 (2022): 1089214. 36618930 10.3389/fphar.2022.1089214 PMC9810635

[36] L. R. Cataldo , N. Vishnu , T. Singh , et al., “The MafA‐Target Gene PPP1R1A Regulates GLP1R‐Mediated Amplification of Glucose‐Stimulated Insulin Secretion in β‐Cells,” Metabolism 118 (2021): 154734. 33631146 10.1016/j.metabol.2021.154734

[37] D. Garcia and R. J. Shaw , “AMPK: Mechanisms of Cellular Energy Sensing and Restoration of Metabolic Balance,” Molecular Cell 66, no. 6 (2017): 789–800. 28622524 10.1016/j.molcel.2017.05.032 PMC5553560

[38] J. Liu , G. Lang , and J. Shi , “Epigenetic Regulation of PDX‐1 in Type 2 Diabetes Mellitus,” Diabetes, Metabolic Syndrome and Obesity 14 (2021): 431–442. 10.2147/DMSO.S291932 PMC7866918 33564250

[39] S. Ferber , A. Halkin , H. Cohen , et al., “Pancreatic and Duodenal Homeobox Gene 1 Induces Expression of Insulin Genes in Liver and Ameliorates Streptozotocin‐Induced Hyperglycemia,” Nature Medicine 6, no. 5 (2000): 568–572. 10.1038/75050 10802714

[40] H. Kaneto , T. Miyatsuka , Y. Fujitani , et al., “Role of PDX‐1 and MafA as a Potential Therapeutic Target for Diabetes,” Diabetes Research and Clinical Practice 77, no. 3 Supple (2007): S127–S137. 17449132 10.1016/j.diabres.2007.01.046

[41] T. Kondo , I. El Khattabi , W. Nishimura , et al., “p38 MAPK Is a Major Regulator of MafA Protein Stability Under Oxidative Stress,” Molecular Endocrinology 23, no. 8 (2009): 1281–1290. 19407223 10.1210/me.2008-0482 PMC2718751

[42] I. El Khattabi and A. Sharma , “Preventing p38 MAPK‐Mediated MafA Degradation Ameliorates β‐Cell Dysfunction Under Oxidative Stress,” Molecular Endocrinology 27, no. 7 (2013): 1078–1090. 23660596 10.1210/me.2012-1346 PMC3706838

[43] H. Chen , S. Brahmbhatt , A. Gupta , and A. C. Sharma , “Duration of Streptozotocin‐Induced Diabetes Differentially Affects p38‐Mitogen‐Activated Protein Kinase (MAPK) phosphorylation in Renal and Vascular Dysfunction,” Cardiovascular Diabetology 4, no. 1 (2005): 3. 15748291 10.1186/1475-2840-4-3 PMC555576

[44] M.‐J. Stahnke , C. Dickel , S. Schröder , et al., “Inhibition of Human Insulin Gene Transcription and MafA Transcriptional Activity by the Dual Leucine Zipper Kinase,” Cellular Signalling 26, no. 9 (2014): 1792–1799. 24726898 10.1016/j.cellsig.2014.04.006 PMC5006626

[45] Y. I. Kitamura , T. Kitamura , J.‐P. Kruse , et al., “FoxO1 Protects Against Pancreatic β Cell Failure Through NeuroD and MafA Induction,” Cell Metabolism 2, no. 3 (2005): 153–163. 16154098 10.1016/j.cmet.2005.08.004

[46] T. Zhang , D. H. Kim , X. Xiao , et al., “FoxO1 Plays an Important Role in Regulating β‐Cell Compensation for Insulin Resistance in Male Mice,” Endocrinology 157, no. 3 (2016): 1055–1070. 26727107 10.1210/en.2015-1852 PMC4769368

[47] M. S. Savova , L. V. Mihaylova , D. Tews , M. Wabitsch , and M. I. Georgiev , “Targeting PI3K/AKT Signaling Pathway in Obesity,” Biomedicine & Pharmacotherapy 159 (2023): 114244. 36638594 10.1016/j.biopha.2023.114244

[48] E. Hajduch , G. J. Litherland , and H. S. Hundal , “Protein Kinase B (PKB/Akt)—A Key Regulator of Glucose Transport?” FEBS Letters 492, no. 3 (2001): 199–203. 11257494 10.1016/s0014-5793(01)02242-6

[49] M. Kobayashi , O. Kikuchi , T. Sasaki , et al., “FoxO1 as a Double‐Edged Sword in the Pancreas: Analysis of Pancreas‐ and β‐Cell‐Specific FoxO1 Knockout Mice,” American Journal of Physiology Endocrinology and Metabolism 302, no. 5 (2012): E603–E613. 22215655 10.1152/ajpendo.00469.2011

